# Exploring Sex-Associated Systemic Inflammatory Profiles Linked to Macrophage Responses and Survival in Pancreatic Ductal Adenocarcinoma

**DOI:** 10.1021/acs.jproteome.6c00739

**Published:** 2026-10-13

**Authors:** Bilal Alashkar Alhamwe, Christian Preusser, Viviane Ponath, Aditya M. Bhagwat, Johannes Graumann, Elke Pogge von Strandmann

**Affiliations:** † Institute for Tumor Immunology, Philipps University Marburg, 35043 Marburg, Germany; ‡ EV-iTEC Core Facility, Philipps University Marburg, 35043 Marburg, Germany; § Institute of Translational Proteomics & Core Facility Translational Proteomics, Philipps University Marburg, 35043 Marburg, Germany

**Keywords:** PDAC, plasma proteomics, tumor-immune environment, sex-specific differences, macrophages

## Abstract

Sex differences influence clinical outcome and therapeutic response in pancreatic ductal adenocarcinoma (PDAC), yet the contribution of systemic immune-inflammatory programs remains insufficiently defined. In this study, we characterized sex-associated circulating proteomic signatures, evaluated their prognostic relevance, and assessed their association with macrophage polarization. Olink Explore quantified 1536 proteins in a cohort of 50 PDAC patients (27 women and 23 men). Proteomic differences were explored alongside TCGA-PAAD survival associations and macrophage responses to conditioned supernatants from male- and female-origin PDAC cell lines. Exploratory analysis identified 51 proteins with nominal sex-associated differences (*p* < 0.05), of which 37 were higher in women and 14 in men. Female-associated proteins were enriched in immune-regulatory and macrophage-associated pathways. Among the PDAC cell lines examined, male-origin supernatants induced M2-like macrophage polarization, whereas female-origin supernatants promoted M1-associated programs. Survival analysis revealed 29 prognostic factors, including 18 sex-specific associations. A female-specific lipid–inflammation module comprising PCSK9, OLR1, IL1RN, and MMP8 was associated with overall survival. Collectively, these findings suggest sex-associated systemic immune-inflammatory profiles link to macrophage responses and prognosis in PDAC, supporting consideration of sex in biomarker development and therapeutic stratification.

## Graphical abstract

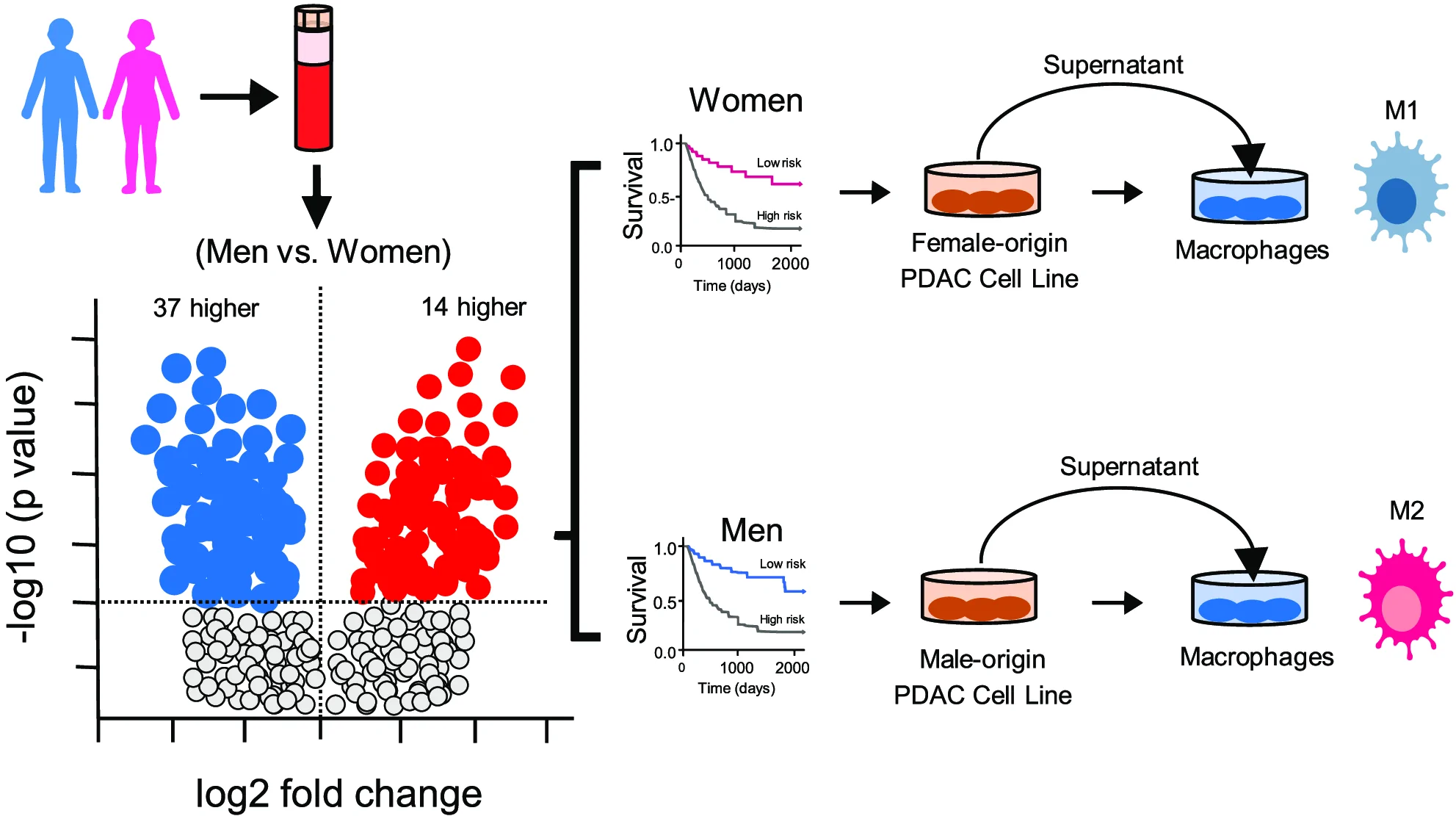

## Introduction

Pancreatic ductal adenocarcinoma (PDAC) has the worst prognosis of all solid tumors, with a global five-year survival rate of approximately 8% and a median survival of about six months after diagnosis.^1^ The incidence rates are rising in industrialized countries, and PDAC is now the second leading cause of cancer death after lung cancer. The main treatment options include surgery, chemotherapy, radiation therapy, and targeted therapy.^2^ However, these approaches are often not curative, and only a fraction of patients respond, highlighting the need for early diagnosis and individualized therapeutic strategies. PDAC progression is shaped not only by malignant tumor cells but also by a highly inflammatory and immunosuppressive tumor microenvironment (TME), which promotes tumor growth while limiting effective antitumor immunity.^3^ A high degree of heterogeneity, with multiple subgroups defined by distinct molecular and cellular characteristics, contributes to diverse clinical outcomes and therapeutic responses.^4–6^

Sex has increasingly emerged as an important biological variable influencing cancer immunity, disease progression, and the therapeutic response. In PDAC, sex differences have also been associated with the clinical outcome. Women show modestly improved overall survival in population-based cohorts, particularly in younger age groups (<50 years),^7,8^ and imaging-based analyses suggest sex-specific correlations between tumor metabolism, morphology, and survival.^9^ At the biological level, variations in immune composition, hormonal regulation, and metabolic parameters may influence tumor behavior and therapy response.^10^

Differences in immune cell infiltration and activity within the TME may, therefore, contribute to sex-dependent clinical outcomes. Supporting this concept, women with fewer tumor-associated macrophages show improved recurrence-free survival following neoadjuvant chemotherapy,^11^ and macrophage infiltration negatively correlates with response to gemcitabine-based chemoradiation in women but not in men.^12,13^ However, whether systemic immune-inflammatory mediator profiles differ between sexes and contribute to these effects remains insufficiently defined.

Here, we used high-dimensional plasma proteomics to link sex-associated systemic protein patterns in treatment-naïve patients with PDAC and investigated their potential biological and clinical relevance. We further examined whether tumor cell sex-of-origin differentially influences macrophage polarization and evaluated the prognostic relevance of the identified sex-biased factors using TCGA-PAAD. Together, this integrated proteomic, functional, and clinical approach was designed to determine whether sex-dependent systemic inflammatory programs represent a biologically relevant component of PDAC.

## Experimental Section

### Patient Cohort and Plasma Samples and Ethical Approval

Human plasma samples were obtained from the Comprehensive Biobank Marburg (CBBMR) following written informed consent from all participants and approval by the Ethics Committee of Philipps University Marburg (reference number 76/17 and subsequent amendments), with consent for the use of anonymized clinical data, including age, sex and survival. All plasma samples were obtained from treatment-naïve PDAC patients. Female and male PDAC patients did not differ significantly in age at diagnosis (66.1 ± 10.9 vs 67.0 ± 7.5 years, respectively; *p* = 0.71, Welch’s *t* test), demonstrating comparable baseline demographic characteristics between the two groups. These plasma samples were collected between 2017 and 2021, aliquoted (1 mL), and stored at −80 °C. Aliquots used for Olink analysis were thawed for the first time at analysis, with no prior freeze–thaw cycles.

Blood samples from healthy adult volunteers were provided by the Center for Transfusion Medicine and Hemotherapy at the University Hospital Giessen and Marburg (UKGM) under separate ethical approval (reference number 205/10, amendment 5), those samples were used for macrophage isolation.

### Olink Explore Proteomic Profiling

Plasma protein profiling was performed using the proximity extension assay (PEA) on the Olink Explore platform, enabling quantification of 1536 proteins. The PEA technology is based on dual antibody recognition, in which matched antibody pairs conjugated to DNA oligonucleotides generate a unique DNA barcode upon target binding, followed by amplification and next-generation sequencing. Plasma samples were thawed on ice, cleared by centrifugation, and incubated with antibody probe pairs according to the manufacturer’s instructions. Library preparation, amplification, and sequencing were performed at the Genomics Core Facility, Philipps University Marburg.

Sequencing reads were processed using the vendor-provided pipeline, including demultiplexing and normalization, based on internal controls. Protein abundance was reported as normalized protein expression (NPX) values on a log_2_ scale. Quality control included filtering based on the detection limits and assay performance metrics. The processed NPX data set was used for downstream analyses (Table S1).

### Bioinformatic and Statistical Analysis

Differential protein abundance between female and male PDAC patients was assessed by using multivariate and univariate approaches. Given the exploratory and hypothesis-generating nature of the study and the limited cohort size, differential protein abundance was assessed using a nominal significance threshold of *p* < 0.05 without correction for multiple testing. Proteins meeting this threshold were considered exploratory candidate sex-associated proteins and subsequently evaluated for functional relationships and pathway enrichment.

Partial least-squares (PLS) analysis was used as an exploratory multivariate approach to visualize overall patterns in the proteomic data set and potential separation between female and male samples, rather than as a predictive classification model. Differentially abundant proteins were visualized using volcano plots and heat maps. Functional enrichment and pathway analysis were performed using STRING network analysis and Gene Ontology annotation. Proteins exhibiting nominal sex-associated differences were subsequently evaluated for functional relationships and enrichment of biological processes. For macrophage experiments, statistical comparisons were performed using the nonparametric Kruskal–Wallis test. Only statistically significant differences are shown. Statistical significance was determined as *p* < 0.05 unless otherwise indicated.

### PDAC Cell Culture and Conditioned Supernatants

To determine whether sex-associated systemic differences might be accompanied by tumor cell-intrinsic differences in immune-modulatory activity, conditioned supernatants were generated from PDAC cell lines derived from male patients (PANC-1 [ATCC CRL-1469; RRID] and MIA PaCa-2 [ATCC CRL-1420; RRID]) or female patients (BxPC-3 [ATCC CRL-1687; RRID] and AsPC-1 [ATCC CRL-1682; RRID]). PANC-1 and MIA PaCa-2 cells (all tested negative for mycoplasma) were cultured in Dulbecco’s Modified Eagle Medium (DMEM; Gibco, NY, USA), whereas BxPC-3 and AsPC-1 cells were cultured in RPMI-1640 (Gibco). The culture media were supplemented with 10% fetal bovine serum (FBS; Gibco), l-glutamine, and penicillin/streptomycin. Cells were seeded at 7 × 10^6^ cells per T175 culture flask in 20 mL of the respective complete culture medium and cultured under hypoxic conditions (1% O_2_, 5% CO_2_, 37 °C) using a Thermo Scientific Heracell VIOS trigas CO_2_ incubator (Thermo Fisher Scientific). After 24 h, the culture medium was completely replaced with fresh medium, and the cells were maintained under hypoxic conditions for 5 days.

Conditioned supernatants were then collected and centrifuged at 300*g* for 10 min at 4 °C to remove cells and cellular debris. The cleared supernatants were harvested for subsequent experiments. All cell lines were routinely tested for mycoplasma contamination before use. The resulting conditioned supernatants were subsequently used to investigate their effects on primary human macrophage polarization.

### Primary Human Macrophage Isolation and Differentiation

Primary human peripheral blood mononuclear cells (PBMCs) were isolated from leukocyte reduction system (LRS) chambers by Ficoll density-gradient centrifugation. Monocytes were enriched by adherence and differentiated into macrophages.

Macrophages were seeded at a density of approximately 2 × 10^5^ cells/well and cultured in RPMI-1640 supplemented with 5% human AB serum and 1% sodium pyruvate in a 12-well plate. After 6 h, medium was replaced with conditioned supernatants (1 mL) derived from PDAC cell lines or control medium.

To assess the impact of tumor cell sex-of-origin, macrophages were exposed to conditioned supernatants from male-derived PDAC cell lines (PANC-1 and MIA PaCa-2) or female-derived cell lines (BxPC-3 and AsPC-1). Macrophages were cultured with conditioned supernatants for 5 days and subsequently analyzed for expression of M1- and M2-associated markers.

### Quantitative RT-PCR

Total RNA was isolated from macrophages after 5 days using the RNeasy Plus Kit (Qiagen) according to the manufacturer’s instructions. Complementary DNA (cDNA) was synthesized using the RevertAid First Strand cDNA Synthesis Kit (Thermo Fisher Scientific). Quantitative real-time PCR (qPCR) was performed using an Agilent Mx3000 system. Gene expression levels were normalized to the housekeeping gene *RPL32*, and relative expression was calculated using the ΔΔ*C_t_* method. Primer sequences are listed in Table S2.

### TCGA-PAAD Survival Analysis

To assess the clinical relevance of the sex-biased circulating proteins, genes corresponding to the 51 nominally sex-associated proteins were evaluated using the TCGA pancreatic adenocarcinoma (TCGA-PAAD, *n* = 185) data set. Survival associations were evaluated separately in female (*n* = 83) and male (*n* = 102) patients, with an age range of 57–73 years. Gene/transcript expression was summarized using the median where multiple measurements were available. Expression was dichotomized into high- and low-expression groups using an optimized survival cut point determined with surv_cut point (maximally selected rank statistics), with the minimum group proportion adjusted according to cohort size. If the optimized cut point could not be determined or resulted in fewer than five patients in either group, median expression was used as the fallback cutoff.

Kaplan–Meier analysis was performed in sex-stratified cohorts to compare overall survival between high- and low-expression groups, with differences assessed using the log-rank test. Cox proportional hazards regression was used to estimate hazard ratios (HRs), 95% confidence intervals, and Wald-test *p*-values. For visualization and classification of survival-associated factors, HRs were log_2_-transformed, with log_2_(HR) > 0 corresponding to HR > 1 and log_2_(HR) < 0 corresponding to HR < 1. *P*-values from the Cox regression and Kaplan–Meier/log-rank analyses were nominal and were not adjusted for multiple testing.

Sex-specific signatures were generated from proteins showing both nominal sex-associated differential abundance in the Olink analysis (*p* < 0.05) and a nominally significant association with overall survival in the corresponding sex-stratified TCGA-PAAD analysis (*p* < 0.05).

Sex-associated signatures were additionally evaluated across the opposite-sex cohort to determine whether their prognostic associations were sex-restricted.

## Results and Discussion

### Sex-Specific Circulating Proteomic Signatures in PDAC

We first investigated whether systemic proteomic profiles differed between female and male patients with PDAC. Consistent with previous reports,^7,8^ female patients in our cohort showed a trend toward improved overall survival compared with male patients (*p* = 0.06; Figure 1A).

**Figure 1:**
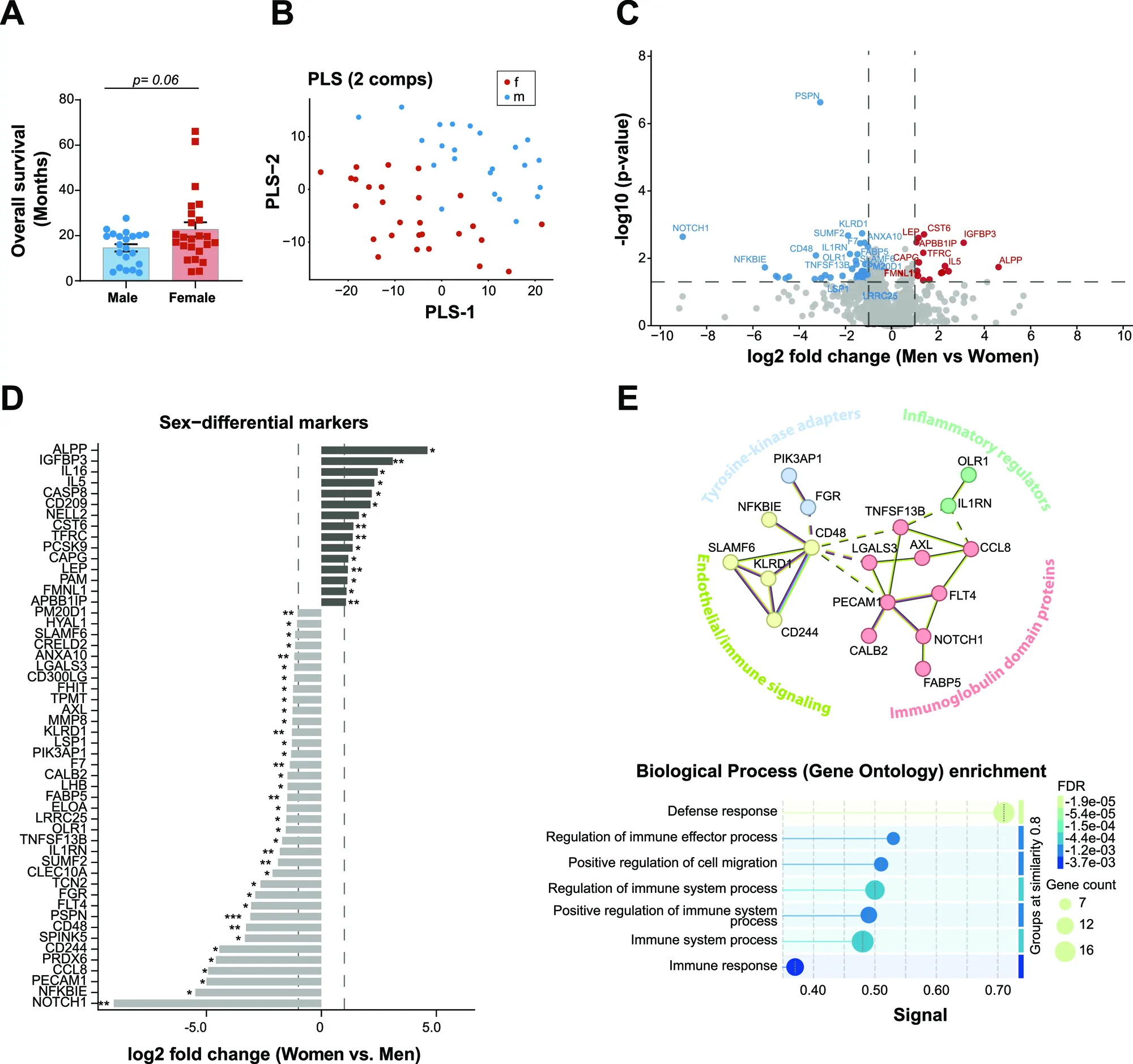
Expression level of circulating proteins in PDAC patients. (A) Survival after diagnosis (months) of the patient cohort stratified into male and female patients. (B) PLS (Partial Least-Squares Regression) analysis of the Olink protein expression data for male and female samples. (C) Volcano plot of detected proteins, male versus female subcohorts. (D) List of all proteins significantly more abundant in male (black) or female (gray) samples (log_2_ fold change >1.5 and *p*-value <0.05). (E) STRING network interaction analysis (https://string-db.org) of selected proteins and gene enrichment analysis for proteins more abundant in female samples (proteins from male patients yielded no results). RStudio was used for statistics and data visualization. *p < 0.05; **p < 0.01; ***p < 0.001.

To determine whether circulating immune-inflammatory mediators differ between sexes, we quantified 1536 plasma proteins by using the Olink Explore platform. Partial least-squares analysis revealed partial separation between female and male patients (Figure 1B), indicating an exploratory visualization of sex-associated variation in the overall plasma proteomic profiles. Exploratory differential abundance analysis identified 51 proteins with nominal sex-associated differences (*p* < 0.05), of which 37 showed higher abundance in female patients and 14 in male patients (Figure 1C,D). STRING network analysis demonstrated clustering of nominally female-associated proteins in immune-regulatory and inflammatory pathways (Figure 1E). Gene Ontology enrichment highlighted immune-related processes, including defense response, immune response, and regulation of immune effector processes.

In contrast, nominally male-associated proteins did not form a dominant pathway cluster, although several including CAPG, TFRC, CST6, IGFBP3, and ALPP have been implicated in PDAC biology and tumor–stroma interactions.^3^ The absence of a dominant cluster may partly reflect the smaller number of male-associated proteins available for enrichment analysis. Several nominally female upregulated proteins, including NFKBIA/NFKBIE, CCL8, LGALS3, CLEC10A, and FGR (Figure 1E), have been linked to macrophage-related signaling and immune polarization in cancer.^14^

Collectively, these findings suggest sex-associated differences in the circulating PDAC proteome, with female patients displaying a particularly prominent enrichment of immune-regulatory and inflammatory factors. These findings are consistent with sex-dependent differences in systemic innate immune regulation. Previous studies have reported that gemcitabine-based neoadjuvant chemoradiation is associated with improved prognosis in women with low levels of tumor-infiltrating M2 macrophages,^12,13^ supporting a role for macrophage-linked inflammation in sex-dependent clinical outcomes.

### Sex-of-Origin-Dependent Macrophage Polarization

We next asked whether the sex-associated differences identified in the circulating proteome could be accompanied by differences in the immune-modulatory activities of PDAC cells. Supernatants from male-derived PDAC cell lines induced higher expression of M2-associated markers CD163 and CD206 compared with control conditions and macrophages exposed to female-derived supernatants (Figure 2A).

**Figure 2:**
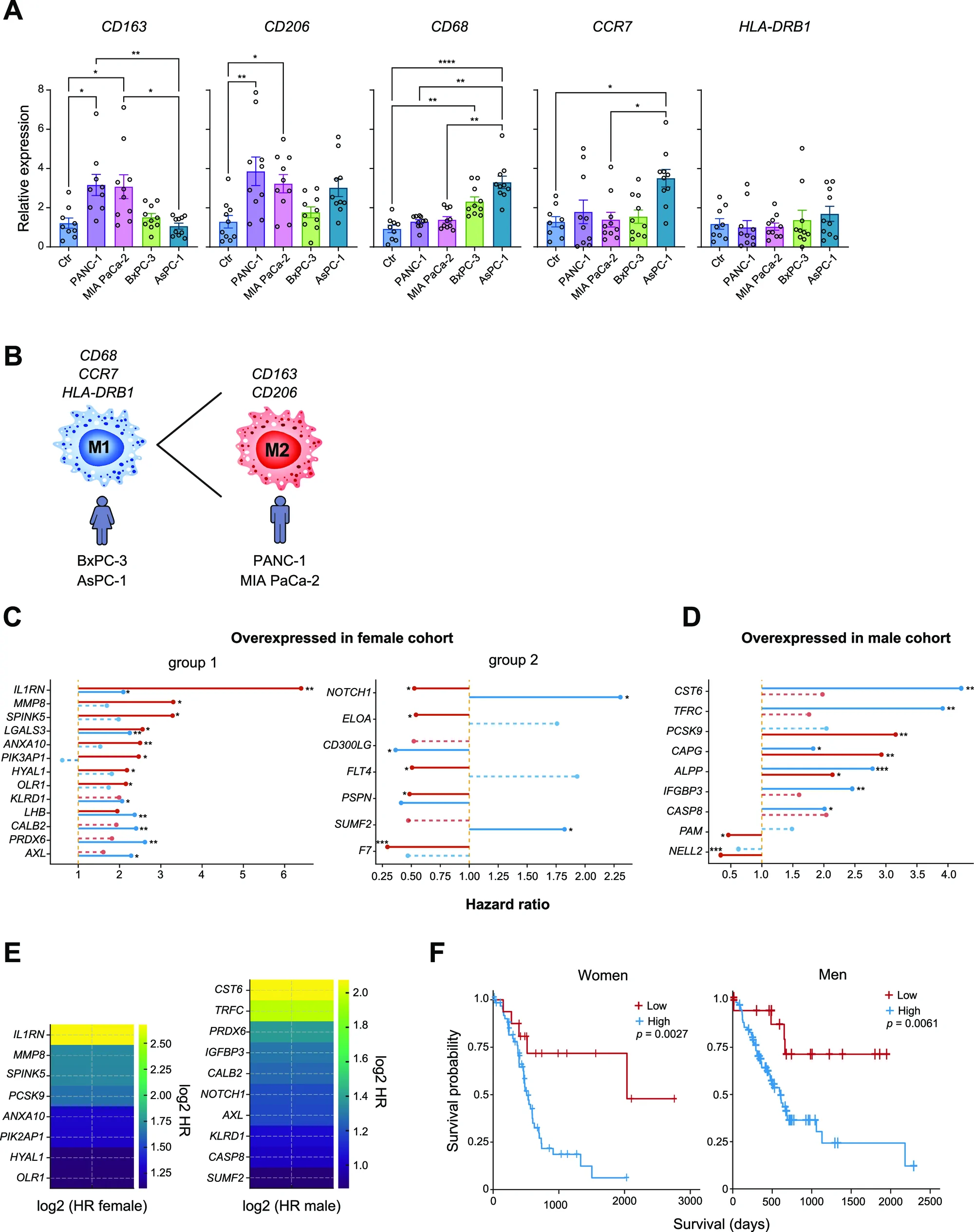
Sex-of-origin effects on macrophage polarization and survival-associated circulating factors. Primary human macrophages derived from healthy donors were cultured for 5 days with conditioned supernatants from PDAC cell lines derived from male patients (PANC-1, MIA PaCa-2) or female patients (BxPC-3, AsPC-1). (A) Relative expression of M2-associated markers (*CD163*, *CD206*) and M1-associated markers (*CD86*, *CCR7*, *HLA-DRB1*) was assessed by quantitative gene expression analysis. (B) A schematic depicts the balance between M1 and M2 macrophage polarization induced by conditioned supernatants from male- or female-derived PDAC cell lines. Lollipop (C, D) and heat map (E) for all factors correlating significantly with survival in the female or male subcohorts (TCGA data set, https://portal.gdc.cancer.gov/projects/TCGA-PAAD). Female and male survival-associated factors were divided into 2 groups according to the direction of the log_2_-transformed hazard ratio, with log_2_(HR) > 0 corresponding to HR > 1 and log_2_(HR) < 0 corresponding to HR < 1 (for clarity). Survival male: blue lines; survival female: red lines; dashed lines indicate nonsignificant *p*-values. (F) Kaplan–Meier analysis of gene signatures depicted in panel (E). RStudio was used for statistics and data visualization. *p < 0.05; **p < 0.01; ***p < 0.001. Data in panel (A) are shown as (mean ± SEM; *n* = 9–10). Each point represents one donor. Statistical significance was determined using the Kruskal–Wallis test; only statistically significant differences are indicated. *p < 0.05; **p < 0.01; ****p < 0.0001.

In contrast, supernatants from female-derived PDAC cell lines preferentially upregulated M1-associated markers CD68 and CCR7, whereas HLA-DRB1 showed a less consistent response (Figure 2A). Thus, PDAC cell lines of male and female origin differentially program macrophage polarization toward M2- or M1-associated phenotypes, respectively (Figure 2B). These in vitro findings parallel the sex-associated differences observed in systemic inflammatory signatures and support an association between macrophage programming and tumor cell sex-of-origin.

Although these experiments do not establish that the circulating proteins identified by Olink directly mediate macrophage polarization, they provide supportive evidence that sex-of-origin-associated differences in PDAC cells can translate into distinct effects on innate immune cell states.

### Sex-Specific Prognostic Protein Signatures

We next investigated whether the sex-biased factors identified by plasma proteomics were associated with clinical outcome. To evaluate clinical relevance, genes corresponding to the 51 sex-biased proteins were analyzed in TCGA-PAAD. Kaplan–Meier analysis identified 29 factors significantly associated with overall survival (Figure 2C,D). Of these, 18 exhibited sex-specific associations, including eight restricted to women and ten to men (Figure 2E).

Kaplan–Meier curves for sex-specific gene signatures demonstrated significant survival stratification within each sex (Figure 2F). These signatures were not predictive when applied across sexes (female signature in men, *p* = 0.25; male signature in women, *p* = 0.19), indicating that sex-specific inflammatory programs may not be captured in nonstratified analyses.

These findings further suggest that sex stratification can reveal prognostic associations that may be obscured when female and male patients are analyzed together. Nevertheless, because the TCGA analysis is based on transcript abundance rather than matched circulating protein measurements, these associations should be interpreted as supportive clinical evidence rather than direct validation of plasma biomarkers.

### Female-Specific Lipid–Inflammation Module

Among the factors associated with survival exclusively in female patients, a lipid–inflammation module comprising PCSK9, OLR1, IL1RN, and MMP8 was identified (Figure 2E). PCSK9 and OLR1 are involved in lipid metabolism and have been linked to NF-κB signaling.^15,16^ Oxidized LDL/OLR1 and PCSK9-dependent pathways can promote PI3K–AKT signaling, linking lipid regulation to inflammatory responses. IL1RN and MMP8 further connect this module to inflammatory regulation and tissue remodeling. These findings suggest that survival in female PDAC patients may be influenced by a systemic lipid–inflammation axis. In contrast, several male-associated factors, including CST6, NOTCH1, and AXL (Figure 2E), have been linked to tumor progression and poor prognosis.^17–19^

Notably, the potential relationship between this lipid–inflammation module and macrophage biology is supported by previous mechanistic observations. PCSK9 has been reported to promote M1 and suppress M2 macrophage polarization in malignancies,^20^ while OLR1 (LOX-1) knockdown reduces M1 polarization in THP-1-derived macrophages.^21^

This module links lipid metabolism to inflammatory signaling and NF-κB-associated pathways and may represent a mechanistic interface between metabolic inflammation and tumor-promoting signaling. Given the central role of myeloid inflammation and immune suppression in PDAC, sex-dependent systemic mediators may influence immune composition, therapy response, or metastatic behavior, although further mechanistic validation will be required.

Taken together, the proteomic, survival, and macrophage-polarization data support a model in which sex is associated with distinct systemic inflammatory states in PDAC, with lipid–inflammatory pathways representing a particularly prominent feature of the female-associated signature.

## Conclusions

This study provides exploratory plasma proteomic evidence for sex-associated differences in systemic immune-inflammatory mediator profiles in PDAC. Exploratory differential abundance analysis identified 51 proteins with nominal sex-associated differences, and proteins showing higher abundance in female patients clustered in immune-regulatory pathways, including proteins associated with innate immune signaling and macrophage-related inflammatory programs. Importantly, survival analyses identified nonoverlapping, sex-specific signatures, supporting the concept that systemic inflammatory biology differs between women and men with PDAC and may contribute to differential prognosis. A notable observation was the identification of a female-specific lipid–inflammation module (PCSK9, OLR1, IL1RN and MMP8) associated with survival.

Beyond these systemic proteomic associations, our functional experiments provide evidence that the male- and female-derived PDAC cell lines examined differ in their effects on macrophage programming. Male-derived PDAC cell lines preferentially induced M2-associated macrophage markers, whereas female-derived cell lines promoted M1-associated programs. These observations are consistent with sex-of-origin-associated differences in tumor-immune crosstalk but do not establish biological sex as the determinant of macrophage polarization.

Macrophages represent a dominant immune population in PDAC and play a central role in shaping both the local and systemic inflammatory states. Their functional plasticity, broadly described along M1- and M2-associated polarization programs, critically influences immune suppression, tumor progression, and therapeutic response.^22^ Tumor- and stroma-derived signals actively reprogram macrophage phenotypes in PDAC, with M2-skewed programs linked to poor outcome and therapy resistance.^12^

Within this study, we identified exploratory sex-associated circulating proteomic patterns in women versus men and demonstrated that many candidate biomarkers show sex-specific associations with overall survival that are obscured in sex-aggregated analyses. Together, these findings highlight sex as a biologically relevant variable in PDAC-associated inflammation with implications for immune stratification and therapeutic targeting.

This study has limitations, including the relatively small cohort size, which does not allow complete adjustment for potential clinical confounders, including tumor stage and comorbidities. In addition, the proteomic analysis was exploratory and based on nominal *p*-values without correction for multiple testing. The identified sex-associated proteins should therefore be considered hypothesis-generating candidates and require validation in larger independent cohorts. Furthermore, plasma measurements provide an indirect readout of tumor biology, and the survival association analysis relies on transcript-level data from TCGA-PAAD rather than matched plasma proteomics.

Furthermore, the macrophage experiments were performed using a limited panel of PDAC cell lines and, therefore, cannot establish that the observed effects are universally determined by biological sex. Because sex-of-origin is confounded with individual cell line identity, the observed differences may also reflect cell line-specific characteristics. Future validation using larger panels, patient-derived models, and direct testing of candidate circulating mediators is important.

Accordingly, future studies integrating tumor tissue analyses and in vivo models will be required to determine how sex-associated macrophage programming contributes to disease progression and treatment response alongside longitudinal integration of systemic inflammatory signatures with tumor-immune infiltration and clinical parameters. In conclusion, our findings support sex as an essential stratification variable in PDAC immunology research and highlight systemic inflammatory networks, including macrophage-associated programs, that may contribute to sex-dependent clinical outcomes.

In conclusion, our findings establish sex as an important stratification variable in PDAC proteomics and identify distinct systemic inflammatory networks with functional and prognostic associations. Integration of sex-stratified proteomics with immune functional analyses may therefore provide a more precise framework for understanding PDAC heterogeneity and for identifying sex-informed biomarkers and therapeutic targets.

## Supplementary Material



## Data Availability

The raw Olink proteomics data, including the raw counts, are available in Table S3.

## Notes

Processed Olink proteomics: Olink Explore NPX values from the PDAC patient cohort (Table S1); primer sequences: Primer sequences used for quantitative RT-PCR analyses (Table S2); and raw Olink proteomics data: Raw Olink counts from the PDAC patient cohort (Table S3) (XLSX)
